# Shifts in the microbiota associated with male mosquitoes (*Aedes aegypti*) exposed to an obligate gut fungal symbiont (*Zancudomyces culisetae*)

**DOI:** 10.1038/s41598-020-69828-9

**Published:** 2020-07-30

**Authors:** Jonas Frankel-Bricker

**Affiliations:** Department of Biological Sciences, Boise State University, Boise, ID 83725 USA

**Keywords:** Applied microbiology, Metagenomics, Microbial ecology, Microbiome, Fungal biology

## Abstract

Research characterizing arthropod-associated microbiota has revealed that microbial dynamics can have an important impact on host phenotypic traits. The influence of fungi on these interactions are emerging as targets for research, especially in organisms associated with global human health. A recent study demonstrated colonization of a widespread gut fungus (*Zancudomyces culisetae*) in a larval mosquito (*Aedes aegypti*) digestive tract affected microbiomes in larvae and newly emerged adult females (Frankel-Bricker et al. Appl Environ Microbiol, 2020. 10.1128/AEM.02334-19) but did not investigate these processes in males. The objective of the study presented here was to assess fungal influences on adult male mosquito microbiomes to enable a more complete assessment of fungal–bacterial–host interactions in the *A. aegypti*–*Z. culisetae* system. Sequencing of 16S rRNA gene amplicons from microbiomes harbored in adult males directly after emerging from pupae revealed larval fungal exposure significantly decreased overall microbial community diversity, altered microbiome composition and structure, and decreased within-group microbiome variation across individuals. Further, bacteria in the family *Burkholderiaceae* were present in high abundance in fungal-exposed males, likely contributing to the disparate microbiota between treatment groups. Comparisons between male and the female microbiomes analyzed in Frankel-Bricker et al. (2020), showed distinct shifts in bacterial communities incurred by larval exposure to fungi, potentially revealing sex-specific fungal–bacterial–host dynamics in *A. aegypti*. These findings highlight the complex role a gut fungus can play in influencing the microbial communities harbored in an important insect and emphasize the significance of accounting for an organism’s sex when studying fungal–bacterial–host dynamics.

## Introduction

Mosquitoes harbor communities of microbes that impact host phenotypic traits^1,2^. Studies investigating factors that contribute to microbial community dynamics provide valuable biological insights into these insects of global significance. Research analyzing mosquito-associated microbiota often investigate bacteria harbored in females^1–11^, in part, due to the distinct capacity of adult females to contract and transmit (vector competence) a variety of human pathogens. Importantly, studies show that certain microbes influence adult female-pathogen interactions^12–16^ and alter vector competence^17–19^, demonstrating how host-microbe interactions affect the proliferation of mosquito-borne diseases. While further analyses of female microbiomes are essential, studies of male-associated microbiota are also necessary. Males have the capacity to transmit certain viruses to females via venereal transmission^20,21^, demonstrating complex, sex-specific host–pathogen interactions occur in this system. Research investigating factors influencing male-associated microbiota are necessary to provide complete assessments of these intricate dynamics.

Several studies comparing male- and female-associated microbiota have revealed distinct characteristics of bacterial communities between the sexes^22,23^. These differences are potentially influenced by disparate ecological behaviors^24,25^, environmental microbiota^9,26–28^, and sources of nutrients^11,29^. In addition, unique microbiomes in sex-specific anatomical regions may also contribute to these divergent microbial community profiles^30^. Male testes and female ovaries harbor similar microbial taxa; however, these microbiomes have different distribution structures^31,32^. Further, the same bacteria exhibit distinct interactions dependent on their presence in either the testes or ovaries^33^. These sex-specific dynamics are also affected by temporal factors during larval development^34^, suggesting unique features of these organs, such as morphological or physiological characteristics, influence certain microbial interactions. Importantly, large amounts of bacteria are expelled from the larval digestive tract during and after pupation^35,36^, indicating the initial microbiota inherited by newly emerged adults originate from other anatomical regions^37,38^ and are transmitted transstadially (from larvae to adults). Differences in the microbiota harbored in adult males and females may be driven by disparate microbiomes harbored in the developing larval sex organs prior to pupation. If this is the case, factors that impact larval microbiomes may have downstream, sex-specific effects on the adult microbiota.

Biological factors such as fungal–bacterial–host interactions are known to affect host-associated microbiomes^39^. Fungi are found in natural mosquito populations^4,7,40^ and impact host fitness^41–43^. Recently, it was demonstrated that a mosquito-associated gut fungus, *Zancudomyces culisetae*, significantly affected microbiome dynamics in larvae and female adults of the yellow fever mosquito (*Aedes aegypti*)^44^. The fungus is an obligate gut fungal symbiont of larval *A. aegypti* and several other dipteran hosts in nature^45,46^, and its life cycle is bound to that of its larval host. Larvae ingest fungal spores from the aquatic environment, fungal growth in the larval hindgut is mediated by specific physiological cues^47,48^, and the fungus acquires nutrients from the host digestive tract^45^. Larval fungal colonization was found to reduce the variation of bacterial community diversity and distribution across individuals, differentially affect the transference efficacy of certain bacterial taxa from larvae to adults (transstadial transmission), and increase the overall diversity of initial microbiomes acquired by newly emerged adult females. Although complex fungal–bacterial–host interactions were revealed, the study did not investigate whether similar dynamics took place in male mosquitoes. A key interpretation of these patterns was that morphological (expansion of fungal tissue) and physiological (altered nutrient dynamics in the digestive tract) disturbances resulting from fungal colonization of the larval hindgut^47,48^ could have altered microbial interactions and spatial distributions of bacteria^49^, leading to differential transstadial transmission outcomes of certain bacteria and disparate initial adult female microbiomes in the presence or absence of the fungus. Male-associated microbial communities may respond differently to fungal colonization of the larval digestive tract due to unique anatomical characteristics, however, this concept has never been studied.

To further investigate fungal–bacterial–host dynamics in the *A. aegypti*–*Z. culisetae* system, comparative analyses were performed on a previously collected but unpublished dataset of sequenced 16S rRNA gene amplicons from microbiomes extracted from newly emerged adult male mosquitoes that developed from larvae with and without exposure to the fungus. I hypothesized that larval exposure to the fungus would alter adult male microbiomes and predicted that these microbial communities would increase in overall diversity and decrease in variation across individuals as previously shown in females. Additionally, I also predicted that different bacterial taxa would be impacted by the fungus due to the known differences between male- and female-associated microbiota.

In this study, the effect of larval fungal exposure on newly emerged adult male microbiomes was determined. These results were compared to those previously found for females, and potential processes contributing to the observed patterns are discussed.

## Results

### Fungal effect on overall microbiome diversity

**Figure 1:**
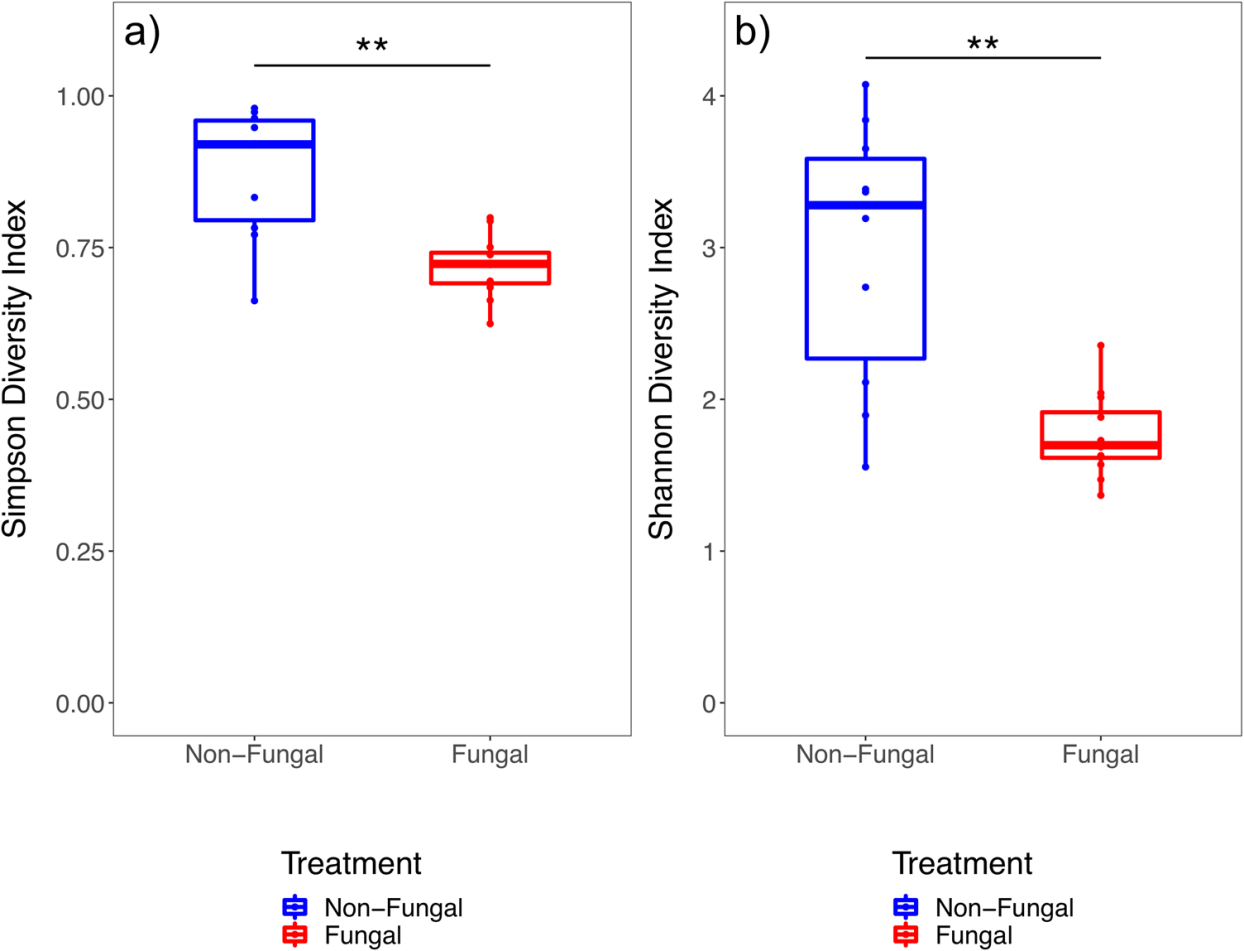
Box plots of alpha diversity measures calculated for microbiomes harbored in newly emerged males developed from larvae in the absence (non-fungal, blue) or presence (fungal, red) of fungal exposure during larval development. (**a**) Comparative analysis of Simpson diversity index shows significantly higher values for non-fungal relative to fungal mosquito microbiomes. (**b**) Comparative analysis of Shannon diversity index shows significantly higher values for non-fungal relative to fungal mosquito microbiomes. Upper and lower limits of boxes represent quartiles around the mean and horizontal lines within boxes represent median values within each treatment group. Significant differences of mean alpha diversity measures were calculated with a linear mixed effects model (*N* = 22, **P < 0.01).

 Measures of alpha diversity were calculated for initial microbiomes to assess whether larval fungal exposure affected the overall diversity of microbial communities acquired by newly emerged adult males (Table S1). Significantly lower mean values were calculated for fungal mosquitoes for both Simpson (*P* < 0.01, Fig. 1a) and Shannon (*P* < 0.01, Fig. 1b) diversity indices, indicating larval exposure to the fungus decreased the overall diversity of microbial communities acquired by newly emerged adults. No significant difference was detected in the variation of alpha diversity measures for either metric (Table 1), however, the CV value for Shannon diversity was higher in non-fungal (29.09%) than fungal (15.47%) groups with *P* < 0.1 calculated with an asymptotic and MSLRT, suggesting a marginal reduction of variation in overall microbial community diversity.

**Table 1:** Results from comparative statistical analyses for measures of alpha and beta diversity across treatment groups.

Metric	DF Num	DF Den	F value	*P* (treatment)	Non-fungal CV	Fungal CV	Test statistic (asymptotic)	CV *P* (asymptotic)	Test statistic (MSLRT)	CV *P* (MSLRT)	DF Num	DF Den	F value	*P* (homogeneity)
Simpson	1	5.967	14.489	**0.009**	12.28%	7.05%	3.009	0.083	2.459	0.117				
Shannon	1	5.975	14.457	**0.009**	29.09%	15.47%	3.598	0.058	3.187	0.074				
Bray–Curtis	1	14.000	6.591	**0.027**							1	20	135.560	**< 0.001**
weighted UniFrac	1	14.000	12.635	**0.027**							1	20	60.766	**< 0.001**
Jaccard	1	14.000	5.449	**0.027**							1	20	74.719	**< 0.001**
Unweighted UniFrac	1	14.000	2.811	**0.043**							1	20	0.040	0.863

Significant differences of mean alpha diversity measures were calculated with linear mixed effects models, beta diversity measures with nested permutational analysis of variance, and within-group variations of beta diversity measures with permutational statistical tests for the homogeneity of group dispersions. Values in boldface are statistically significant (*P* < 0.05). These data are presented and described following the format provided in Frankel-Bricker et al.^44^.

### Distinct microbiome composition and structure across treatments

**Figure 2:**
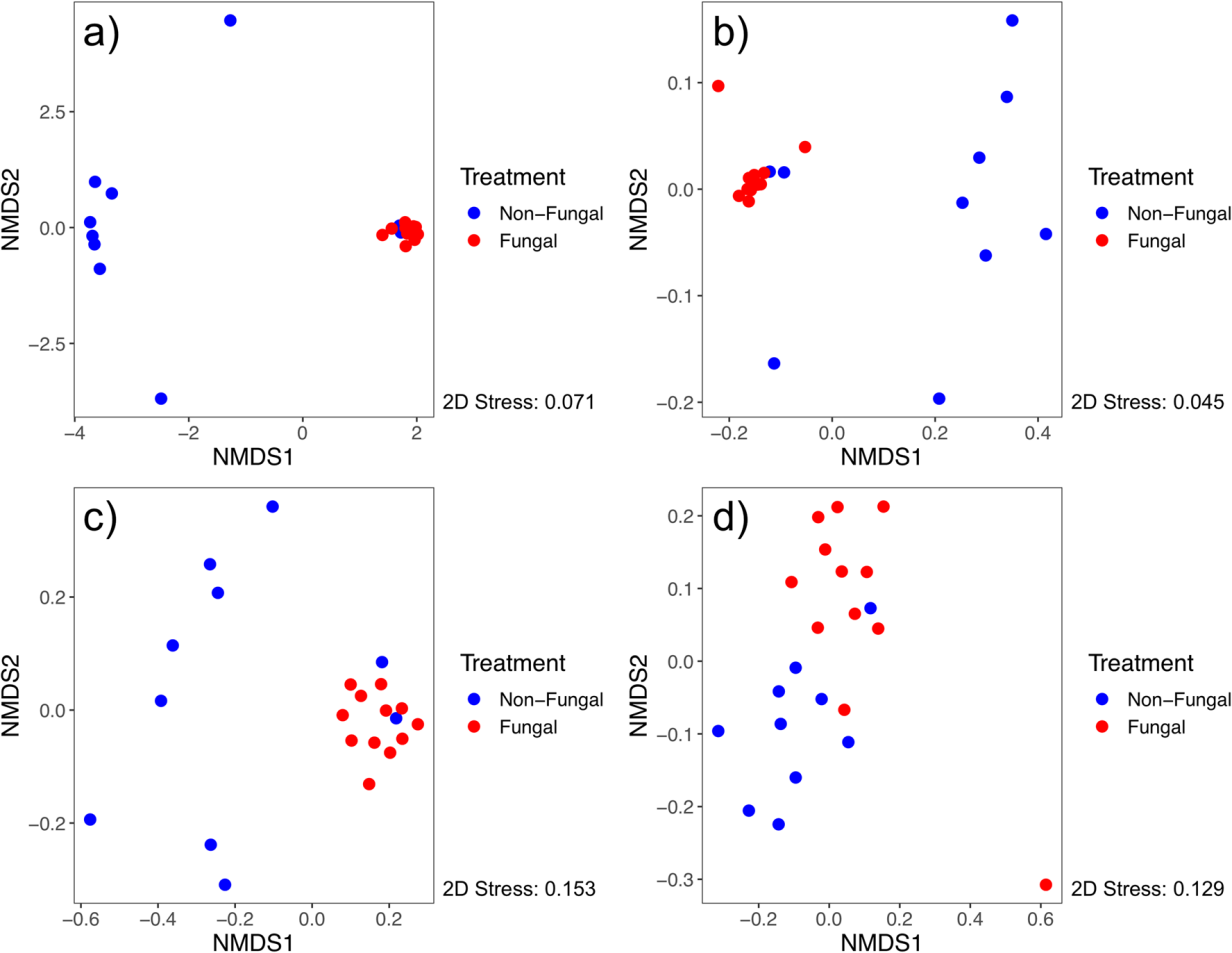
Nonmetric multidimensional scaling plots of beta diversity measures across treatment groups (non-fungal, blue; fungal, red) for (**a**) Bray–Curtis dissimilarity, (**b**) weighted UniFrac distance, (**c**) Jaccard index, and (**d**) unweighted UniFrac distance.

**Figure 3:**
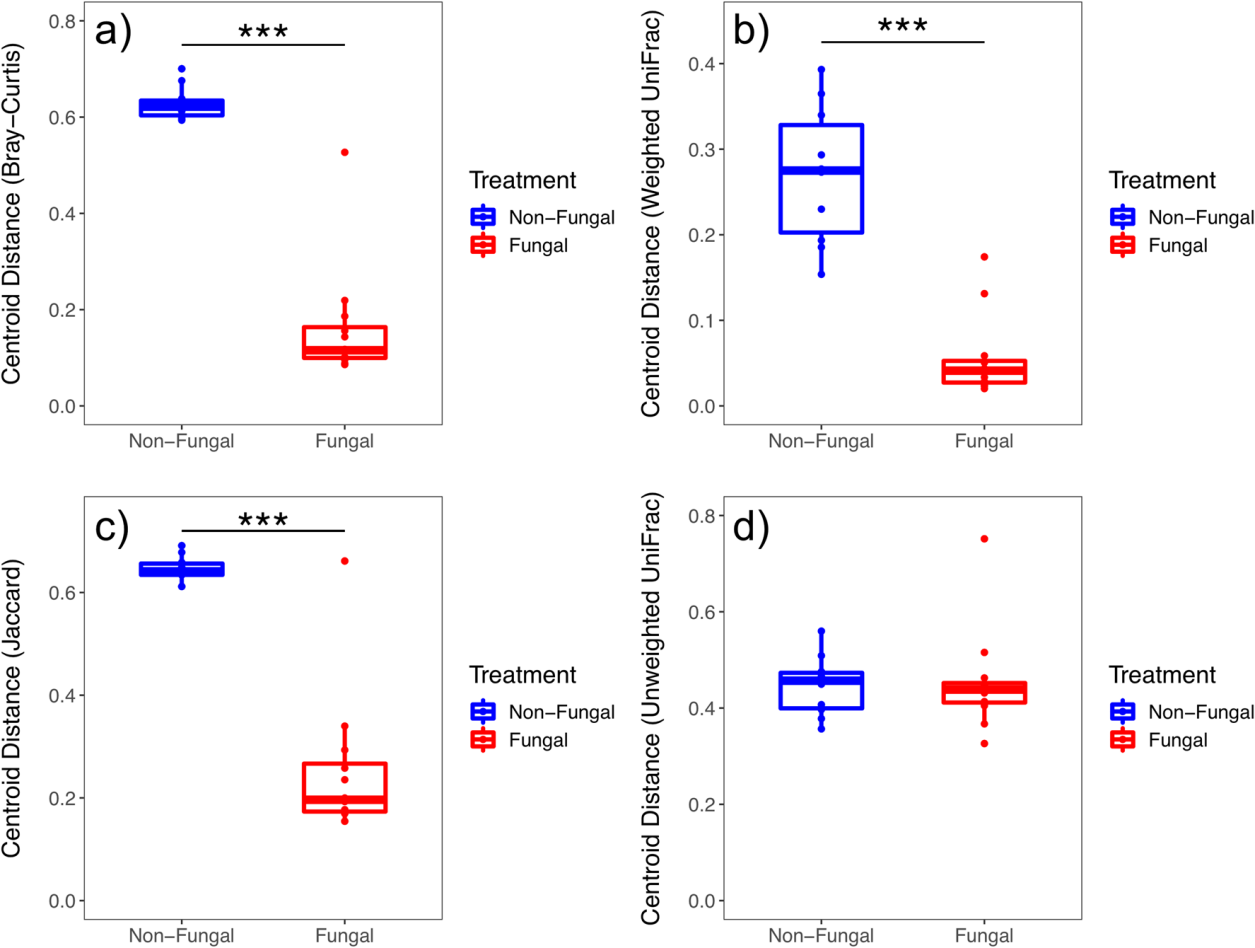
Box plots of within treatment group variation of beta diversity measures for (**a**) Bray–Curtis dissimilarity, (**b**) weighted UniFrac distance, (**c**) Jaccard index, and (**d**) unweighted UniFrac distance. Significant differences were calculated with permutational statistical tests for the homogeneity of group dispersions (***P < 0.001).

After read processing, 444 ASVs were identified from 22 samples. Differences in beta diversity measures across treatment groups were detected with PERMANOVA for all metrics tested (Table 1), showing larval exposure to the fungus contributed to disparate microbial communities acquired by newly emerged adults. Permutational analysis of variance tests are sensitive to differences in variation across group comparisons and may produce false detections of significance, however, NMDS plots show separation between treatment groups for all metrics tested (Fig. 2). Non-fungal mosquitoes had higher within-group variation for Bray–Curtis dissimilarity (*P* < 0.001, Fig. 3a), weighted UniFrac distance (*P* < 0.001, Fig. 3b), and Jaccard index (*P* < 0.001, Fig. 3c), indicating the fungus reduced structural variation of the microbiota across individuals in the group. However, no differential within-group variation was detected for unweighted UniFrac distance (*P* = 0.863, Fig. 3d), demonstrating taxonomic composition variation of the microbial communities was not affected by the fungus. These significant patterns were reproduced by analyses conducted on a non-rarefied data set (Table S2), demonstrating that the implementation of rarefaction did not affect these results.

**Figure 4:**
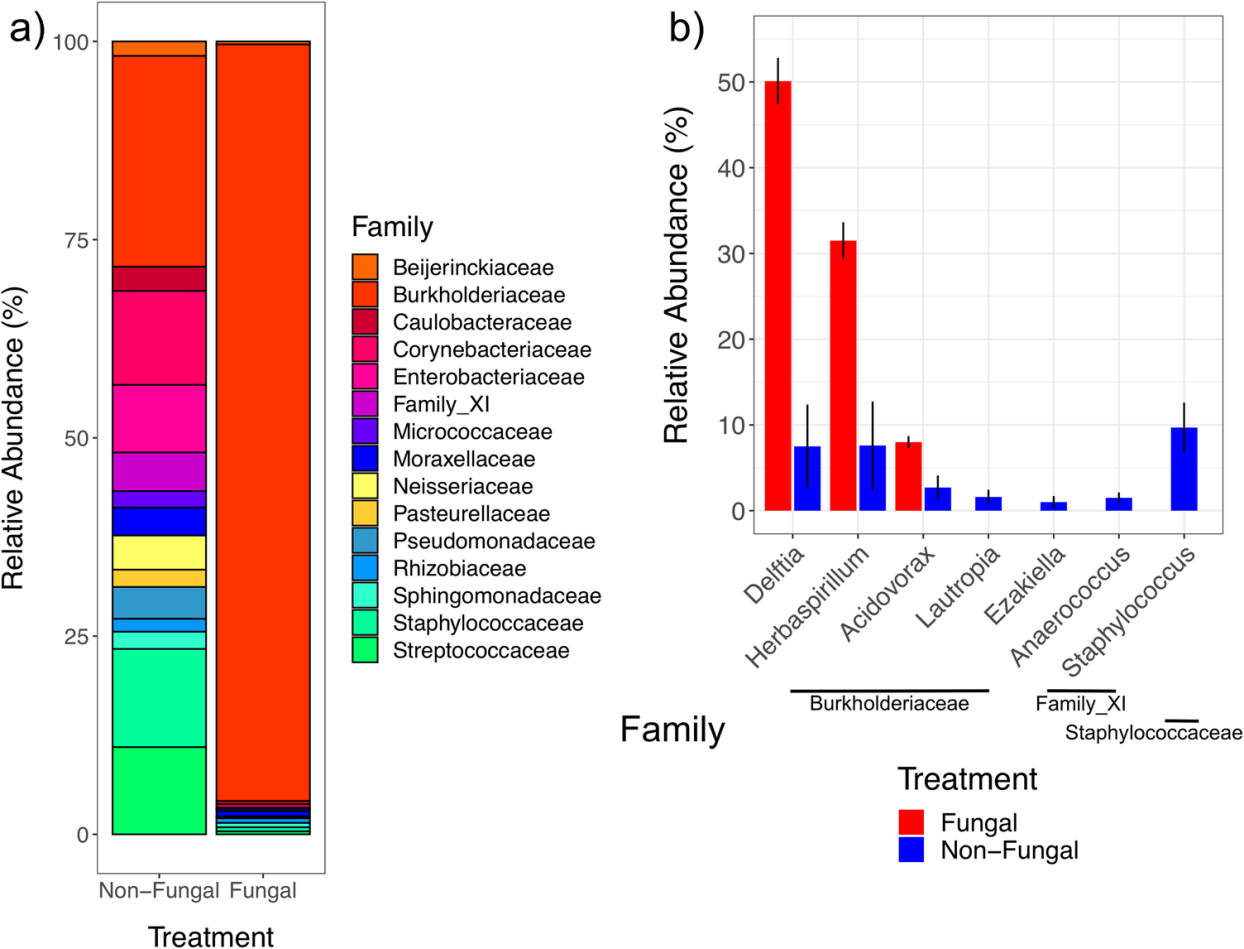
Mean relative abundances of prominent bacterial taxa across treatment groups. (**a**) Bar plots of the 15 most abundant families shared between treatment groups. (**b**) Bar plots of genera within families identified to significantly differ across treatment groups. Only genera present at greater than 1% relative abundance within each treatment group are shown. Line segments represent standard errors of the mean.

Relative abundances of bacterial families were calculated to investigate whether certain taxa were differentially impacted by the fungus (Fig. 4a, Table S3). The bacterial family *Burkholderiaceae* was present at moderate levels in non-fungal adults (21.06%) but was significantly more abundant in fungal adults (89.75%, *P* < 0.01). Conversely, *Staphylococcaceae*, an intermediate community member in non-fungal adults (9.81%), was found at significantly lower levels in fungal adults (0.48%, *P* = 0.013). *Family_XI* followed a similar trend and was present at low amounts in non-fungal (3.91%) but was nearly undetectable in fungal adults (0.01%, *P* = 0.036). These significant families were further analyzed at the genus level to assess whether genera abundances followed similar patterns within families (Fig. 4b; Table S4). Within *Burkholderiaceae*, three of the four predominant genera detected had higher abundance in fungal relative to non-fungal adults (*Delftia*, *Herbaspirillum*, *Acidovorax*), whereas the genus *Staphylococcus* (*Staphylococcaceae*) was higher in non-fungal adults, demonstrating genera followed similar trends as their associated families.

### Supplementary analyses of female-associated microbiomes

Measures of alpha diversity were calculated for initial microbiomes harbored in newly emerged females and compared with previously published findings^44^ (Table S1; Fig. S1) to assess general similarities or differences between the frozen samples analyzed here and those not frozen in the earlier study. Measures calculated in the current experiment fell within the previously calculated ranges for non-fungal mosquitoes and in the lower ranges of fungal mosquitoes for Simpson and Shannon diversity indices. General trends were conserved, with non-fungal mosquitoes having larger variation than fungal mosquitoes for both metrics (Fig. S1) and similar mean values across groups. Female sample sizes collected for the current study were small (n = 9) and did not represent all replicate containers in the experiment, preventing a more in-depth statistical analysis of these data. However, the detection of similar trends for females between the two experiments for alpha diversity measures could indicate a reproducible result and suggests comparisons between these experiments may be appropriate.

## Discussion

The results presented herein provide additional evidence that a widespread gut fungus played a significant role in its mosquito host’s microbiome. Substantial differences in the composition and structure of bacterial communities harbored across treatment groups were found, supporting the hypothesis that larval fungal exposure would impact initial adult male microbiomes. Fungal microbiomes were dominated by bacteria in the family *Burkholderiaceae*, with nearly 90% of sequencing reads assigned to genera within this family (*Delftia* [50.1%], *Herbaspirillum* [31.5%], *Acidovorax* [8.0%]). The differential abundance of these microbes likely defined the overall characteristics and differences in microbiome composition and structure. Fungal mosquitoes had significantly lower overall diversity than non-fungal mosquitoes (Fig. 1) and different taxonomic composition and community structure (Figs. 2, 4; Table 1). Low within-group variation of beta diversity measures for fungal mosquitoes (Fig. 3) indicated that the high relative abundance of *Burkholderiaceae* was a conserved characteristic of these microbial communities. While this bacterial family was present at intermediate levels in non-fungal adults (21.1%), it was more evenly distributed with the other families and corresponded with higher community diversity, reflected by significantly higher values for Simpson (Fig. 1a) and Shannon (Fig. 1b) diversity indices (Table S1). Morphological and physiological characteristics of fungal colonization in the larval hindgut^47,48,50^ may have driven these disparities in the microbiota. The spatial distribution of fungal tissue, physiological shifts in the digestive tract microecosystem^47,48^, and depletion of nutrients from the host digestive tract incurred by the fungus^45^ could have promoted the proliferation of *Burkholderiaceae* if taxa in this family were better adapted to these fungal-induced changes^49,51^. Future research could investigate these concepts through inoculation experiments of larvae with experimentally controlled microbiota to assess whether similar taxa persist after larval fungal colonization. In addition, studies of these dynamics could be performed on wild-caught mosquitoes to assess whether the patterns described here are conserved in nature.

Comparisons with previous findings for females^44^ uncovered possible evidence of sex-specific fungal–bacterial–host dynamics in the system. I originally predicted that shifts in the initial male microbiome in response to larval fungal exposure would follow similar patterns as those previously described in females, which were characterized by lower within-group variation but similar means for alpha diversity measures relative to non-fungal adults. Contrary to this prediction, within-group variation in alpha diversity measures for male samples did not statistically differ across treatments and non-fungal males harbored microbiomes with significantly higher overall diversity relative to fungal males (Fig. 1; Table 1). Further, strong differences in microbiome composition and structure were detected across treatments in males (Figs. 2, 3) that were not previously found in females. These differences could reflect sex-specific fungal–bacterial–host dynamics, possibly driven by the influence of the fungus on the transmission of bacterial taxa in the family *Burkholderiaceae*. Adult females were previously shown to have higher prevalence of *Burkholderiaceae* in the absence of larval fungal colonization. Conversely, adult males from the fungal group were dominated by this taxon, possibly suggesting differential sex-specific interactions between the fungus and these bacteria (Fig. 4). Spatial disturbances in the larval hindgut during fungal colonization and expansion^50^ may have redistributed the microbiota. Indeed, fungal displacement of microbial communities was observed in a pathogenic mosquito-fungus system and impacted host fitness^43^. Mosquitoes expel large quantities of bacteria from the digestive tract during and after pupation^35,36^, indicating that bacteria inherited in newly emerged adults may be transferred transstadially through anatomical regions other than the digestive tract^37,38^. It is possible that the differential abundance of *Burkholderiaceae* in newly emerged adult males is driven by these mechanisms. Male and female reproductive organs harbor microbiomes composed of similar bacterial taxa but with different community distributions^31,32^. In the absence of fungal colonization, microbiota in the testes may be characterized with relatively high overall diversity. During larval fungal infestation, bacteria may spatially redistribute to the testes, or to other anatomical regions, altering existing microbial interactions and resulting in favorable conditions for genera within *Burkholderiaceae*. Different processes related to unique microbiomes in female ovaries may have led to distinct microbial dynamics and resulted in the disparate patterns between fungal male and female microbiomes. Future studies could perform experiments to investigate the spatial distribution of bacteria in the family *Burkholderiaceae* in males and females prior to and after pupation in the presence and absence of larval fungal colonization by utilizing previously described fluorescence-based assays^37,38^. Dissections isolating the testes and ovaries could also be performed on larvae throughout development to provide temporal information of these potentially sex-specific microbiomes. Results from these experiments could help clarify the mechanisms leading to differential transstadial transmission outcomes of *Burkholderiaceae*. In addition, future research could investigate whether bacteria in this family impact adult male fitness or have downstream impacts on life history traits. Experiments in this field may reveal distinct, sex-specific microbiome dynamics in mosquito populations contingent on the presence or absence of the fungus in the local environment.

Alpha diversity measures were calculated for a small subset of females to compare with previous findings^44^. Values fell within or near previous calculations and within-treatment variation followed similar trends. Non-fungal mosquitoes exhibited higher within-group variation than fungal mosquitoes, increasing confidence that comparisons of the primary results between experiments are appropriate. However, I acknowledge that the small samples sizes used for comparative analyses of females limits the power of these comparisons. Therefore, a future experiment should be designed to fully analyze the microbiomes harbored in males and females under standardized laboratory protocols and with larger sampling sizes. Reproducibility remains a fundamental, yet elusive element in microbial ecology and microbiome research^52^. Further research should be conducted under independent laboratory conditions to corroborate the results presented herein, as proposed and conducted in human studies and arthropod systems^53–55^.

## Conclusion

Here, a significant effect of larval fungal exposure on host-associated microbiota in newly emerged adult male mosquitoes is revealed, providing further support that fungi are important microbial community members in *A. aegypti*. Preliminary evidence for sex-specific fungal–bacterial–host dynamics in a mosquito-fungus symbiotic system merits further investigation. Comprehensively studying all components of microbiomes is essential to understand biotic interactions contributing to an organism’s biology. These findings suggest studies of host-associated microbiomes should account for the presence of fungi, the host organism’s sex, and potentially distinct sex-specific host-microbe relationships.

## Materials and methods

The majority of the protocols conducted for this experiment were carried out in tandem with those presented in Frankel-Bricker et al.^44^. Consequently, there is extensive overlap in the description of experimental design and protocols.

Male samples evaluated in this experiment were reared and collected alongside the females, however, these were separately frozen prior to DNA extraction and the subsequent sequencing analyses were conducted independently. This difference in collection and storage method may have altered downstream detection of certain bacterial taxa^56,57^, warranting independent analyses for these experimental samples.

### *Zancudomyces culisetae* culture maintenance

A culture of *Z. culisetae* (USDA-ARS Collection of Entomopathogenic Fungal Cultures, Ithaca, New York, USA, ARSEF 9012, *Smittium culisetae*, COL-18-3) was grown at room temperature on a 1/10 brain heart infusion agar plate with 3 milliliters (ml) autoclaved Nanopure water (Barnstead Thermolyne Corp., Dubuque, IA, USA). Antibiotics were added to the overlay to mitigate bacterial contamination of the fungal culture (2 mg/ml of penicillin and 7 mg/ml of streptomycin). The overlay was filtered through a sheet of Miracloth (EMD Millipore, Burlington, MA, USA) and into 1.5 ml microcentrifuge tubes (Eppendorf, Hamburg, Germany) to collect fungal spores. Spores were concentrated by centrifugation at 900 g for 10 minutes (min) and the supernatant was discarded. The resulting pellets were resuspended in 1 ml autoclaved Arrowhead bottled spring water (Nestle, Vevey, Switzerland) and spore concentrations were estimated with a Neubauer Improved C-Chip Hemocytometer (SKC Inc., Covington, GA, USA) visualized with phase optics light microscopy.

### Experimental conditions and mosquito rearing

*Aedes aegypti* eggs, derived from the USDA-ARS Gainesville line (Benzon Research Inc., Carlisle, PA, USA) were stored at room temperature. Histology containers (Fisher Scientific, Pittsburgh, PA, USA) were filled with 350 ml of bottled spring water and subsequently autoclaved. Four containers were assigned to each of two experimental treatment groups: larvae not exposed to fungal spores (Non-Fungal), and larvae exposed to fungal spores (Fungal). Approximately 50 eggs were added to each rearing container, containers were covered with 4 layers of autoclaved Miracloth to mitigate airborne contamination and were separately placed in a vacuum chamber (SP Industries Inc., Warminster, PA, USA) for 30 min to synchronize egg hatch timing^58^. The larval mosquito food source was prepared by finely grinding Tetramin Fish Food (Tetra, Melle, Germany) with a mortar and pestle and suspending 0.2 g of fish food powder per 10 ml autoclaved bottled spring water. One ml of the slurry was added to each rearing container at the start of the experiment. Containers assigned to the fungal group were inoculated with approximately 400,000 fungal trichospores. Mosquitoes were reared at 24 °C ± 1 °C with a 16:8 h light/dark cycle in a low temperature refrigerated incubator (Fisher Scientific, model #3724), and 1–2 ml of fish slurry were added daily to each rearing container. All experimental protocols were performed on a sterilized laboratory workbench next to a Bunsen burner to minimize contamination.

### Mosquito sample collection

Mosquitoes were reared to pupae, transferred to 1.5 ml microcentrifuge tubes, and surface-sterilized following an adult protocol described in Coon et al.^5^. Surface-sterilized pupae were transferred separately to 15 ml centrifuge tubes (Corning Inc., Corning, NY, USA) containing 7 ml autoclaved bottled spring water and reared axenically for 2–3 days until adult emergence. The sex of newly emerged adults was visually identified and individual adult males were transferred to 1.5 ml microcentrifuge tubes and stored at − 80 °C. In addition, 5 adult females were collected from the non-fungal group and 4 from the fungal group and frozen similarly.

### Microbial DNA extraction

The Quick-DNA Fungal/Bacterial Kit (Zymo Research, Irvine, CA, USA) was used to extract microbial DNA from frozen male and female mosquitoes and other experimental sources following the protocol provided by the manufacturer with the following modifications previously described in Frankel-Bricker et al.^44^: Lysis buffer was added directly to the 1.5 ml microcentrifuge tubes containing harvested mosquitoes. Mosquitoes were manually ruptured with an autoclaved pellet pestle (DWK Life Sciences, Wertheim, Germany) for approximately 1–2 min. Homogenized tube mixtures were transferred to bead tubes supplied with the extraction kit and disrupted using a vortex mixer at maximum setting for 5 min. The elution buffer was heated to 45 °C prior to its application to the spin-filters supplied by the extraction kit and remained on the filter surface for 5 min prior to the final elution step. Extracted microbial DNA was stored at − 80 °C.

At least 2 DNA extractions were performed on mosquitoes collected from each replicate container for the non-fungal treatment group (10 total) and 3 from each replicate container for the fungal group (12 total). Extractions were also performed on a small subset of female mosquitoes (9 total) from 5 of the replicate containers (3 non-fungal, 2 fungal). Additional DNA extractions were performed on blank extraction kit reagents from the 2 kits used to account for possible contamination of kit reagents. Two blank polymerase chain reactions (PCR) were also carried out to check for contamination of PCR reagents. All PCR were performed using 5PRIME HotMasterMix (Quantabio, Beverly, MA, USA).

### Bacterial 16S rRNA gene amplicon sequencing

The V3/V4 hypervariable regions of microbial 16S rDNA were amplified with primer pair 341f/785r^59^, with suggested linker sequences^60^, and adapter and spacer sequences provided by the University of Idaho Genomics Resources Core (GRC) (University of Idaho, Moscow, ID, USA). Targeted 16S PCR were performed on extracted experimental DNA samples using four primer pair variants containing spacer sequences of different lengths to mitigate amplification biases (Tables S5, S6: Rxn_1). PCR products were visualized on 1.5% agarose gels to confirm amplification of 16S rDNA.

Secondary PCR were performed (Table S6: Rxn_2) to attach barcode sequences provided by the University of Idaho GRC to PCR amplicons. Amplicon sequencing was performed with an Illumina MiSeq v3 (Illumina Inc., San Diego, CA, USA) at the University of Idaho GRC, producing 300 base pair paired end reads. Reads were demultiplexed by sample barcode sequences by the sequencing facility.

### Raw read processing and ASV assignment

The bioinformatics pipeline and subsequent analyses were performed using the R programming language version 3.6.2^61^. The DADA2 pipeline^62^ was used to process paired end reads. Forward and reverse reads were trimmed to 278 base pairs and 167 base pairs, respectively, and at the location of the first occurrence of a base call with a Phred score less than or equal to 15, filtered by discarding reads with any number of N base calls or containing greater than or equal to 6 estimated errors, and merged with a minimum overlap of 12 bases. Experimental samples with less than 100 reads after initial filtering were removed from the pipeline. Chimeric sequences were discarded and merged reads dereplicated. Taxonomy was assigned to amplicon sequence variants (ASVs) using the SILVA v132 database^63,64^. A neighbor-joining tree was inferred using the *phangorn* package in R^65^ and a generalized time-reversible with gamma rate variation maximum likelihood tree was fit using the neighbor-joining tree as the starting point. The phylogenetic tree, taxonomically assigned ASVs, read count data, and experimental sample metadata were combined into a single data object using the *Phyloseq* package in R^66^.

### Contaminant ASV removal

Non-experimental sources, such as reagents from DNA extraction kits and PCR, can add contaminant sequences and mislead analyses of microbiomes if not properly accounted for^67^. Of the controls sequenced, both extraction kits used had over 100 reads after processing in DADA2. Reads from extraction kits were pooled and the *decontam* package in R^68^ was used to identify contaminant ASVs with the “prevalence” method and the threshold set to 0.5. Amplicon sequence variants classified as likely contaminants originating from the extraction kits were discarded from the sequencing dataset prior to downstream analyses.

### Transformation and analyses of sequencing data

Non-transformed reads were used to calculate Simpson and Shannon alpha diversity indices (Table S1) in *Phyloseq*. Values for female samples were combined with those previously calculated in a larger experiment^44^. Box plots (Fig. S1) were generated using the *ggplot2* package in R^69^. Coefficient of variation values (CV; the ratio of the standard deviation to the mean) were calculated with the *sjstats* package in R^70^ to assess variation of alpha diversity measures, and the R package *cvequality*^71^ was used to calculate significant differences in CV values with an asymptotic test^72^ and a modified signed-likelihood ratio test (MSLRT)^73^. Rarefaction curves were generated using the *ranacapa* package^74^ and *ggplot2* in R (Fig. S2). Read coverage varied across samples (min: 1532 reads; max: 12,420 reads). Sequencing data were rarefied to 1532 reads (the lowest read count for a sequenced sample). Singletons were removed and ASVs not represented by at least 5 reads in one sample after rarefaction were discarded.

Beta diversity measures [Bray–Curtis dissimilarity (compositional dissimilarity), unweighted UniFrac distance (qualitative compositional similarity weighted for phylogenetic distance of taxa), weighted UniFrac distance (quantitative compositional comparison weighted for phylogenetic distance), and Jaccard similarity index (compositional similarity)] were calculated in *Phyloseq* and non-metric multidimensional scaling (NMDS) plots were created in combination with *ggplot2*. Tests for significant differences between treatment groups were performed with permutational analysis of variance (PERMANOVA)^75^ with 999 permutations in the *Vegan* package in R^76^, along with the *nested.npmanova* function in the *BiodiversityR* package in R^77^. Nested PERMANOVA calculated the correct pseudo F and *P* values for the treatment effect while accounting for random effects across replicate rearing containers. Dispersions of beta diversity (the distance from an individual measure to the group’s centroid) were calculated for each beta diversity metric within each group in *Vegan* to estimate within-group variation^78^ across individuals. Significant differences in beta diversity variation were tested using permutational statistical tests for the homogeneity of group dispersions^79^ with 999 permutations in *Vegan* and box plots were constructed in *ggplot2*. These analyses were also performed on a non-rarefied data set to test whether rarefaction impacted results from these tests. Mean relative abundances of the top 15 bacterial families shared between groups (which accounted for greater than 79% of the total reads) were calculated (Table S3) and stacked bar plots were created in *Phyloseq* in combination with *ggplot2*. Relative abundances of genera within significant families were calculated (Table S4) and taxa found at greater than a mean of 1% in a group were plotted using *ggplot2*.

### Linear mixed effects models

The statistical significance of the treatment effect on mean alpha diversity measures and relative abundances of each of the top 15 shared bacterial families were calculated by fitting a linear mixed effects model constructed with the *lme4* package in R^80^, which accounted for random effects across replicate rearing containers. Models were tested with Type II Wald F tests with Kenward-Roger degrees of freedom using the *car* package in R^81^.

### Approval for the use of animal samples

All experimental protocols for the rearing and harvesting of mosquito samples were in strict accordance with the guidelines and regulations set forth and approved by the Boise State University Institutional Biosafety Committee.

## Supplementary information

Supplementary File S1

Supplementary Figures

Supplementary Table S1

Supplementary Table S2

Supplementary Table S3

Supplementary Table S4

Supplementary Table S5

Supplementary Table S6

## Data Availability

Raw sequences are deposited in the NCBI Sequence Read Archive under accession number PRJNA606895.
