# Supplementary material for: Shifts in the microbiota associated with male mosquitoes (Aedes aegypti) exposed to an obligate gut fungal symbiont (Zancudomyces culisetae)
Source: Sci Rep. 2020 Jul 30;10:12886. doi: 10.1038/s41598-020-69828-9 (PMC7393158; doi:10.1038/s41598-020-69828-9)
Supplement: Supplementary file 1 — Supplementary File S1 [file 41598_2020_69828_MOESM1_ESM.pdf]

FileS1

Jonas Frankel-Bricker

2/10/2020

Contents

Packages required **1**

Identification and removal of contaminant ASV sequences **2**

Alpha diversity measurements **2**

Beta diversity measurements **4**

Linear mixed models for relative abundances of top 15 families **6**

Paper figures **7**

Supplemental figures **11**

Packages required

```
# R version v 3.5.1
library(dada2)
  #v 1.8
library(phangorn)
  #v 2.4
library(phyloseq)
  #v 1.24.2
library(genefilter)
  #v 1.62.0
library(tidyverse)
  #v 1.2.1
library(lme4)
  #v 1.1-19
library(car)
  #v 3.0-2
library(cvequality)
  #v 0.1.3
library(sjstats)
  #v 0.17.2
library(plyr)
  #v 1.8.4
library(cowplot)
  #v 0.0.3
library(devtools)
  #v 2.0.1
source_url("https://raw.githubusercontent.com/gauravsk/ranacapa/master/R/ggrare.R")
library(vegan)
```

```
  #v 2.5-3
library(BiodiversityR)
  #2.10-1
library(Rmisc)
  #v 1.5
library(reshape)
  #v 3.5.1
library(decontam)
  #v 3.10
```

Identification and removal of contaminant ASV sequences

```
# Import phyloseq object
pstree <- readRDS("Data/pstree_16S.rds")
meta <- pstree@sam_data

# Extraction kits used for samples
pstree_kit <- subset_samples(pstree, Extraction_Kit == "3" | Extraction_Kit == "4")
meta_kit <- pstree_kit@sam_data

pstree_frozen <- subset_samples(pstree_kit, Frozen == "TRUE" | Type == "kit")
meta_frozen <- pstree_frozen@sam_data

# Identify contaminants with 'prevalence' method and threshold set to 0.5
sample_data(pstree_frozen)$is.neg <- sample_data(pstree_frozen)$Type == "kit"

contam.prev <- isContaminant(pstree_frozen, method = "prevalence", neg = "is.neg",
                             threshold = 0.5)
table(contam.prev$contaminant)
FALSE  TRUE
2246     9

# Prune contaminant ASVs from dataset
pstree_frozen_decom <- prune_taxa(!contam.prev$contaminant, pstree_frozen)
```

Alpha diversity measurements

```
# Subset for males
pstree_males <- subset_samples(pstree_frozen_decom, sex == "Males")

### Alpha diversity calculations
set.seed(192387)

meta_males <- pstree_males@sam_data
richness_males <- data.frame(estimate_richness(pstree_males, split = TRUE,
                                               measures = NULL))
richness_males$Sample_ID <- rownames(richness_males)
alpha_males <- left_join(meta_males, richness_males)
```

```r
# Linear mixed effects models
males_nonfungal <- subset(alpha_males, Treatment == "Non-Fungal")
males_fungal <- subset(alpha_males, Treatment == "Fungal")

### Simpson
male_Simpson_lmer <- lmer(data = alpha_males, Simpson ~ Treatment +
                            (1 | Source_Pop), REML = TRUE)
car::Anova(male_Simpson_lmer, test.statistic = "F")
Response: Simpson
              F Df Df.res   Pr(>F)
Treatment 14.489  1 5.9674 0.008993 **

# Coefficient of Variation
cv(males_nonfungal$Simpson, males_fungal$Simpson)
0.12278719 0.07049415

# Asymptotic test
asymptotic_test(alpha_males$Simpson, alpha_males$Treatment)
$D_AD
3.008949

$p_value
0.08280596

# MSLRT
mslr_test(nr = 1000, alpha_males$Simpson, alpha_males$Treatment)
$MSLRT
2.459133

$p_value
0.1168432


### Shannon
male_Shannon_lmer <- lmer(data = alpha_males, Shannon ~ Treatment +
                            (1 | Source_Pop), REML = TRUE)
car::Anova(male_Shannon_lmer, test.statistic = "F")
Response: Shannon
              F Df Df.res   Pr(>F)
Treatment 14.457  1 5.9752 0.009015 **

#Coefficient of Variation
cv(males_nonfungal$Shannon, males_fungal$Shannon)
0.2908956 0.1547289

# Asymptotic test
asymptotic_test(alpha_males$Shannon, alpha_males$Treatment)
$D_AD
3.598388

$p_value
0.05783562
```

```r
# MSLRT
mslr_test(nr = 1000, alpha_males$Shannon, alpha_males$Treatment)
$MSLRT
3.187096

$p_value
0.07422174
```

#Rarefaction and filtering of reads

```r
#Create sample sums table
males_samsums <- data.frame(sample_sums(pstree_males))
colnames(males_samsums) <- "Read_Counts"

#Rarefy to lowest read count for a sample (1532 reads)
males_rarefy <- rarefy_even_depth(pstree_males, sample.size = 1532)

#Filter out singletons
singfilterfun1 <- filterfun(kOverA(1, 1))
males_filter1 <- filter_taxa(males_rarefy, function(x) singfilterfun1(x), TRUE)

#Convert to relative abundance
males_relative1 <- transform_sample_counts(males_filter1, function(x) x / sum(x))

###Greater than 0.3% in at least 1 occurrence (removes OTUs 5 reads or less)
singfilterfun2 <- filterfun(kOverA(1, 0.003))
males_relative2 <- filter_taxa(males_relative1, function(x) singfilterfun2(x), TRUE)
```

Beta diversity measurements

```r
set.seed(192387)

### Bray-Curtis dissimilarity
df_bray_males <- as(sample_data(males_relative2), "data.frame")
d_bray_males <- distance(males_relative2, "bray")

# Nested Permanova
nested.npmanova(d_bray_males ~ Treatment + Source_Pop, data = df_bray_males,
                permutations = 999, method = "bray")
            Df SumsofSquares      F N.Perm Pr(>F)
Treatment    1        1.9969 6.5911    999  0.027 *
Source_Pop   6        1.8178 1.6338    999  0.049 *
Residuals   14        2.5961 0.1854

# Betadisper
bray_betadisper <- betadisper(d_bray_males, df_bray_males$Treatment)
permutest(bray_betadisper, permutations = 999)
Response: Distances
          Df  Sum Sq Mean Sq    F N.Perm Pr(>F)
Groups     1 1.18963 1.18963 135.56    999  0.001 ***
Residuals 20 0.17551 0.00878
```

```
### Weighted UniFrac
df_wuni_males <- as(sample_data(males_relative2), "data.frame")
d_wuni_males <- distance(males_relative2, "wunifrac")

# Nested Permanova
nested.npmanova(d_wuni_males ~ Treatment + Source_Pop, data = df_wuni_males,
                permutations = 999, method = "wunifrac")
          Df SumsofSquares       F N.Perm Pr(>F)
Treatment   1       0.68421 12.6349    999  0.027 *
Source_Pop  6       0.32491  1.4911    999  0.236
Residuals  14       0.50842  0.0363

# Betadisper
wuni_betadisper <- betadisper(d_wuni_males, df_wuni_males$Treatment)
permutest(wuni_betadisper, permutations = 999)
Response: Distances
          Df   Sum Sq  Mean Sq      F N.Perm Pr(>F)
Groups     1 0.251049 0.251049 60.766    999  0.001 ***
Residuals 20 0.082628 0.004131


### Jaccard
df_jaccard_males <- as(sample_data(males_relative2), "data.frame")
d_jaccard_males <- distance(males_relative2, "jaccard")

# Nested Permanova
nested.npmanova(d_jaccard_males ~ Treatment + Source_Pop, data = df_jaccard_males,
                permutations = 999, method = "jaccard")
            Df SumsofSquares  F     N.Perm Pr(>F)
Treatment   1        1.8020 5.4487    999  0.027 *
Source_Pop  6        1.9844 1.4782    999  0.048 *
Residuals  14        3.1322 0.2237

# Betadisper
jaccard_betadisper <- betadisper(d_jaccard_males, df_jaccard_males$Treatment)
permutest(jaccard_betadisper, permutations = 999)
          Df  Sum Sq Mean Sq     F N.Perm Pr(>F)
Groups     1 0.84830 0.84830 74.791    999  0.001 ***
Residuals 20 0.22684 0.01134


### Unweighted UniFrac
df_uni_males <- as(sample_data(males_relative2), "data.frame")
d_uni_males <- distance(males_relative2, "unifrac")

# Nested Permanova
nested.npmanova(d_uni_males ~ Treatment + Source_Pop, data = df_uni_males,
                permutations = 999, method = "unifrac")
          Df SumsofSquares      F N.Perm Pr(>F)
Treatment   1       0.71084 2.8112    999  0.043 *
Source_Pop  6       1.51715 1.1483    999  0.085 .
Residuals  14       3.08289 0.2202
```

```
# Betadisper
uni_betadisper <- betadisper(d_uni_males, df_uni_males$Treatment)
permutest(uni_betadisper, permutations = 999)
Response: Distances
        Df   Sum Sq   Mean Sq      F N.Perm Pr(>F)
Groups     1 0.000309 0.0003091 0.0395    999  0.863
Residuals 20 0.156441 0.0078221
```

Linear mixed models for relative abundances of top 15 families

```
males_relative2_family <- tax_glom(males_relative2, taxrank = "Family")

# Identify the top 15 shared bacterial families
family_15 <- names(sort(taxa_sums(males_relative2_family), TRUE)[1:15])

# Prune top 15
family_prune <- prune_taxa(family_15, males_relative2_family)
sum(sample_sums(family_prune)) / sum(sample_sums(males_relative2_family))
0.8961138

# Table with top 15 family names
family_table <- cbind(tax_table(family_prune))

# Subset for non-fungal males
nonfungal_males <- subset_samples(males_relative2, Treatment == "Non-Fungal")
# Subset for fungal males
fungal_males <- subset_samples(males_relative2, Treatment == "Fungal")

### Burkholderiaceae

# Non-Fungal
Burkholderiaceae_nonfungal <- subset_taxa(nonfungal_males, Family == "Burkholderiaceae")
# Relative Abundance
sum(sample_sums(Burkholderiaceae_nonfungal)) / nsamples(Burkholderiaceae_nonfungal)
0.2106335

# Fungal
Burkholderiaceae_fungal <- subset_taxa(fungal_males, Family == "Burkholderiaceae")
# Relative Abundance
sum(sample_sums(Burkholderiaceae_fungal)) / nsamples(Burkholderiaceae_fungal)
0.8975037

### Burkholderiaceae LMER

# Combined data
Burkholderiaceae <- subset_taxa(males_relative2, Family == "Burkholderiaceae")
# Metadata sheet
Burkholderiaceae_meta <- Burkholderiaceae@sam_data
# Abundance variable
Burkholderiaceae_meta$Abundance <- sample_sums(Burkholderiaceae)
# Dataframe
```

```
Burkholderiaceae_metadata <- as(Burkholderiaceae_meta, Class = "data.frame")

# Final linear mixed-effects model
Burkholderiaceae_lmer <- lmer(data = Burkholderiaceae_metadata, Abundance ~
                                Treatment + (1 | Source_Pop), REML = TRUE)
Anova(Burkholderiaceae_lmer, test.statistic = "F")
Response: Abundance
            F Df Df.res   Pr(>F)
Treatment 29.706  1 5.9867 0.001598 **

### Repeat workflow for:
Pseudomonadaceae
Enterobacteriaceae
Staphylococcaceae
Rhizobiaceae
Corynebacteriaceae
Streptococcaceae
Caulobacteraceae
Moraxellaceae
Family_XI
Neisseriaceae
Beijerinckiaceae
Pasteurellaceae
Sphingomonadaceae
Micrococcaceae
```

Paper figures

```
### Figure 1

# a (Simpson Diversity)
male_Simpson <- ggplot(alpha_males, aes(Treatment, Simpson, color = Treatment)) +
  geom_boxplot() + theme_bw()

male_Simpson_final <- male_Simpson +
  scale_y_continuous(limits = c(0, 1.1),
                     breaks = c(0, 0.25, 0.5, 0.75, 1)) +
  scale_color_manual(values = c("#0000FF", "#FF0000")) +
  geom_jitter(position = position_jitterdodge(jitter.width = 0)) +
  geom_segment(aes(x = 1, xend = 2, y = 1.05, yend = 1.05), color = "Black") +
  annotate(geom = "text", x = 1.5, y = 1.07, label = "**", color = "black", size = 10) + ylab("Simpson [illegible]
  theme(text = element_text(size = 25),
        axis.title.y = element_text(margin = margin(r = 15)),
        axis.title.x = element_text(margin = margin(t = 15)),
        panel.grid.major = element_blank(),
        panel.grid.minor = element_blank(), panel.border = )

# b (Shannon Diversity)
male_Shannon <- ggplot(alpha_males, aes(Treatment, Shannon, color = Treatment)) +
  geom_boxplot() + theme_bw()
```

```
male_Shannon_final <- male_Shannon + scale_y_continuous(limits = c(0, 4.4),
                                                        breaks = c(0, 1, 2, 3, 4)) + scale_color_manual
  geom_jitter(position = position_jitterdodge(jitter.width = 0)) +
  geom_segment(aes(x = 1, xend = 2, y = 4.25, yend = 4.25), color = "Black") +
  annotate(geom = "text", x = 1.5, y = 4.29, label = "**", color = "black", size = 10) + ylab("Shannon I
  theme(text = element_text(size = 25), axis.title.y = element_text(margin = margin(r = 15)),
        axis.title.x = element_text(margin = margin(t = 15)),
        panel.grid.major = element_blank(), panel.grid.minor = element_blank(),
        panel.border = )


### Figure 2
set.seed(192387)

# a (Bray-Curtis dissimilarity)
males_bray_nmds <- ordinate(males_relative2, "NMDS", "bray")
2D Stress: 0.0706372

males_brayplot_nmds <- plot_ordination(males_relative2, males_bray_nmds, type = "samples",
                                       color = "Treatment") + theme_bw()

males_brayplot_nmds_final <- males_brayplot_nmds +
  scale_color_manual(values = c("#0000FF", "#FF0000")) +
  theme(text = element_text(size = 25), panel.grid.major = element_blank(),
        panel.grid.minor = element_blank(), panel.border = )

# b (weighted UniFrac)
males_wuni_nmds <- ordinate(males_relative2, "NMDS", "wunifrac")
2D Stress: 0.0448847

males_wuniplot_nmds <- plot_ordination(males_relative2, males_wuni_nmds,
                                       type = "samples", color = "Treatment") + theme_bw()

males_wuniplot_nmds_final <- males_wuniplot_nmds + scale_color_manual(values = c("#0000FF", "#FF0000"))
  theme(text = element_text(size = 25), panel.grid.major = element_blank(),
        panel.grid.minor = element_blank(), panel.border = )

# c (Jaccard)
males_jaccard_nmds <- ordinate(males_relative2, "NMDS", "jaccard", binary = TRUE)
2D Stress: 0.1528478

males_jaccard_nmds <- plot_ordination(males_relative2, males_jaccard_nmds,
                                      type = "samples", color = "Treatment") + theme_bw()

males_jaccard_nmds_final <- males_jaccard_nmds +
  scale_color_manual(values = c("#0000FF", "#FF0000")) +
  theme(text = element_text(size = 25), panel.grid.major = element_blank(),
        panel.grid.minor = element_blank(), panel.border = )

# d (unweighted UniFrac)
males_uni_nmds <- ordinate(males_relative2, "NMDS", "unifrac")
2D Stress: 0.1286544
```

```r
males_uniplot_nmds <- plot_ordination(males_relative2, males_uni_nmds,
                                     type = "samples", color = "Treatment") + theme_bw()

males_uniplot_nmds_final <- males_uniplot_nmds +
  scale_color_manual(values = c("#0000FF", "#FF0000")) +
  theme(text = element_text(size = 25), panel.grid.major = element_blank(),
        panel.grid.minor = element_blank(), panel.border = )


### Figure 3

# a (Bray-Curtis dissimilarity)
bray_betadisper_data <- data.frame(bray_betadisper$group, bray_betadisper$distances)
colnames(bray_betadisper_data) <- c("Treatment", "Distance")

betadisper_brayplot <- ggplot(bray_betadisper_data, aes(Treatment, Distance,
                                                        color = Treatment))
+ geom_boxplot() + ylab("Distance from Centroid (Bray-Curtis)") + xlab("Treatment")

betadisper_brayplot_final <- betadisper_brayplot + scale_y_continuous(limits = c(0, 0.8)) + xlab("") + [illegible]
  geom_jitter(position = position_jitterdodge(jitter.width = 0)) +
  geom_segment(aes(x = 1, xend = 2, y = 0.75, yend = 0.75), color = "black") +
  annotate(geom = "text", x = 1.5, y = 0.76, label = "***", size = 10, color = "black") + theme_bw()

betadisper_brayplot_final + theme(text = element_text(size = 25),
                                  axis.title.y = element_text(margin = margin(r = 15)),
                                  panel.grid.major = element_blank(), panel.grid.minor =
                                    element_blank(), panel.border = )

# b (weighted UniFrac)
wuni_betadisper_data <- data.frame(wuni_betadisper$group, wuni_betadisper$distances)
colnames(wuni_betadisper_data) <- c("Treatment", "Distance")

betadisper_wuniplot <- ggplot(wuni_betadisper_data, aes(Treatment, Distance,
                                                        color = Treatment)) +
  geom_boxplot() + ylab("Distance from Centroid (Weighted UniFrac)") + xlab("Treatment")

betadisper_wuniplot_final <- betadisper_wuniplot + scale_y_continuous(limits = c(0, 0.45)) +
  xlab("") + scale_color_manual(values = c("#0000FF", "#FF0000")) +
  geom_jitter(position = position_jitterdodge(jitter.width = 0)) +
  geom_segment(aes(x = 1, xend = 2, y = 0.425, yend = 0.425), color = "black") +
  annotate(geom = "text", x = 1.5, y = 0.435, label = "***", size = 10, color = "black") +
  theme_bw()

betadisper_wuniplot_final + theme(text = element_text(size = 25), axis.title.y =
                                    element_text(margin = margin(r = 15)),
                                  panel.grid.major = element_blank(), panel.grid.minor =
                                    element_blank(), panel.border = )

# c (Jaccard)
jaccard_betadisper_data <- data.frame(jaccard_betadisper$group, jaccard_betadisper$distances)
colnames(jaccard_betadisper_data) <- c("Treatment", "Distance")
```

```
betadisper_jaccardplot <- ggplot(jaccard_betadisper_data, aes(Treatment, Distance,
                                                              color = Treatment)) +
  geom_boxplot() + ylab("Distance from Centroid (Weighted UniFrac)") + xlab("Treatment")

betadisper_jaccardplot_final <- betadisper_jaccardplot +
  ylab("Distance from Centroid (Jaccard)") + scale_y_continuous(limits = c(0, 0.75)) +
  xlab("") + scale_color_manual(values = c("#0000FF", "#FF0000")) +
  geom_jitter(position = position_jitterdodge(jitter.width = 0)) +
  geom_segment(aes(x = 1, xend = 2, y = 0.72, yend = 0.72), color = "black") +
  annotate(geom = "text", x = 1.5, y = 0.725, label = "***", size = 10, color = "black") +
  theme_bw()

betadisper_jaccardplot_final + theme(text = element_text(size = 25),
                                     axis.title.y = element_text(margin = margin(r = 15)),
                                     panel.grid.major = element_blank(), panel.grid.minor =
                                       element_blank(), panel.border = )

# d (unweighted UniFrac)
uni_betadisper_data <- data.frame(uni_betadisper$group, uni_betadisper$distances)
colnames(uni_betadisper_data) <- c("Treatment", "Distance")

betadisper_uniplot <- ggplot(uni_betadisper_data, aes(Treatment, Distance,
                                                      color = Treatment)) +
  geom_boxplot() + ylab("Distance from Centroid (Unweighted UniFrac)") + xlab("Treatment")

betadisper_uniplot_final <- betadisper_uniplot +
  scale_y_continuous(limits = c(0, 0.8)) + xlab("") +
  scale_color_manual(values = c("#0000FF", "#FF0000")) +
  geom_jitter(position = position_jitterdodge(jitter.width = 0)) + theme_bw()

betadisper_uniplot_final + theme(text = element_text(size = 25),
                                 axis.title.y = element_text(margin = margin(r = 15)),
                                 panel.grid.major = element_blank(), panel.grid.minor =
                                   element_blank(), panel.border = )


### Figure 4

# a (family abundance bar plot)

# Identify top 15 shared families
family_15 <- names(sort(taxa_sums(males_relative2_family), TRUE)[1:15])

# Prune top 15
family_15_prune <- prune_taxa(family_15, males_relative2_family)
sum(sample_sums(family_15_prune)) / sum(sample_sums(males_relative2_family))
0.8961138

# Make table
family_15_table <- cbind(tax_table(family_15_prune))

# Consolidate table formatting
family_15_table[family_15, "Family"] <- as(tax_table(family_15_prune)[family_15, "Family"], "character")
```

```r
tax_table(family_15_prune) <- family_15_table

# Transform to percentages
family_15_merge <- merge_samples(family_15_prune, "Treatment")
family_15_percent <- transform_sample_counts(family_15_merge, function(x) 100 * x / sum(x))

# Plot relative abundances of top 15 families
males_barplot <- plot_bar(family_15_percent, fill = "Family")
males_barplot <- males_barplot + xlab("Adult Type") + ylab("Relative Abundance (%)") + theme_bw()

males_barplot <- males_barplot + xlab("Treatment") + scale_x_discrete(limits = c("Non-Fungal", "Fungal")
  scale_fill_manual(values =
                      c("#FF6600", "#FF3300", "#CC0033", "#FF0066", "#FF0099", "#CC00CC",
                        "#6600FF", "#0000FF", "#FFFF66", "#FFCC33", "#3399CC", "#0099FF",
                        "#33FFCC", "#00FF99", "#00FF66"))

males_barplot_final <- males_barplot + theme(text =
                                               element_text(size = 20),
                                             axis.title.y = element_text(margin =
                                                                           margin(r = 15)),
                                             axis.title.x = element_text(margin =
                                                                           margin(t = 15)),
                                             panel.grid.major = element_blank(),
                                             panel.grid.minor = element_blank(),
                                             panel.border = )

# b (genera abundance bar plot)
genera_comb$Abundance <- genera_comb$Abundance * 100
genera_comb_top <- subset(genera_comb, greater_1percent == "TRUE")

genera_abundanceplot <- ggplot(genera_comb_top, aes(Genus, Abundance, fill = Treatment)) +
  geom_bar(stat = "identity", position = position_dodge2(width = 0.5, preserve = "single")) +
  scale_x_discrete(limits =
                     c("Delftia", "Herbaspirillum", "Acidovorax", "Lautropia",
                       "Ezakiella", "Anaerococcus", "Staphylococcus")) + theme_bw()

genera_abundanceplot <- genera_abundanceplot + xlab("") + ylab("Relative Abundance (%)") +
  scale_fill_manual(values = c("#FF0000", "#0000FF")) +
  theme(axis.text.x = element_text(angle = 90, hjust = 1, vjust = 0.5),
        text = element_text(size = 25), axis.title.y = element_text(margin = margin(r = 15)),
        axis.title.x = element_text(margin = margin(t = 15)),
        plot.margin = unit(c(0.5, 0.5, 2, 1), "cm")) + guides(fill = FALSE)
```

Supplemental figures

```r
### Figure S1 (rarefaction curve)
males_rarecurve <- ggrare(pstree_males, step = 100, color = "Treatment", se = FALSE) +
  theme_bw()
males_rarecurve <- males_rarecurve + xlab("Sample Read Coverage") + ylab("ASV Richness")

males_rarecurve_final <- males_rarecurve + scale_color_manual(values = c("#0000FF", "#FF0000")) +
```

```
  geom_vline(xintercept = 1532, linetype = "dashed") +
  xlab("Sample Read Coverage")

males_rarecurve_final + theme(text = element_text(size = 20),
                              axis.title.y = element_text(margin = margin(r = 15)),
                              axis.title.x = element_text(margin = margin(t = 15)),
                              panel.grid.major = element_blank(),
                              panel.grid.minor = element_blank(), panel.border = )


### Figure S2 (female alpha diversity comparisons)

# a (Simpson Diversity Index)
Simpson_Female <- ggplot(Final_Old, aes(Treatment, Simpson, color = Experiment,
                                        fill = Treatment), size = 1) +
  geom_boxplot() + scale_x_discrete(limits = c("Non-Fungal", "Fungal"))

Simpson_Female <- Simpson_Female + geom_boxplot(data = Final_New,
                                                aes(Treatment, Simpson,
                                                    color = Experiment,
                                                    fill = Treatment), size = 1) +
                    scale_x_discrete(limits = c("Non-Fungal", "Fungal")) +
                    scale_y_continuous(limits = c(0, 1),
                                       breaks = c(0, 0.25, 0.5, 0.75, 1.00)) +
                    scale_fill_manual(values = c("#FF0000", "#0000FF")) +
                    scale_color_manual(values = c("#000000", "#CCCCCC")) +
                    xlab("Treatment") +
                    ylab("Simpson Diversity Index") +
                    theme_bw()

Simpson_Female + theme(text = element_text(size = 25),
                       axis.title.y = element_text(margin = margin(r = 15)),
                       axis.title.x = element_text(margin = margin(t = 15)),
                       panel.grid.major = element_blank(),
                       panel.grid.minor = element_blank(), panel.border = )

# b (Shannon Diversity Index)
Shannon_Female <- ggplot(Final_Old, aes(Treatment, Shannon,
                                        color = Experiment, fill = Treatment), size = 1) +
  geom_boxplot() + scale_x_discrete(limits = c("Non-Fungal", "Fungal"))

Shannon_Female <- Shannon_Female + geom_boxplot(data = Final_New,
                                                aes(Treatment, Shannon,
                                                    color = Experiment,
                                                    fill = Treatment), size = 1) +
                 scale_x_discrete(limits = c("Non-Fungal", "Fungal")) +
                 scale_y_continuous(limits = c(0, 4.2), breaks = c(0, 1, 2, 3, 4)) +
                 scale_fill_manual(values = c("#FF0000", "#0000FF")) +
                 scale_color_manual(values = c("#000000", "#CCCCCC")) +
                 xlab("Treatment") +
                 ylab("Shannon Diversity Index") +
                 theme_bw()
```

```r
Shannon_Female + theme(text = element_text(size = 25),
                       axis.title.y = element_text(margin = margin(r = 15)),
                       axis.title.x = element_text(margin = margin(t = 15)),
                       panel.grid.major = element_blank(), panel.grid.minor = element_blank(),
                       panel.border = )
```
