# Supplementary material for: Shifts in the microbiota associated with male mosquitoes (Aedes aegypti) exposed to an obligate gut fungal symbiont (Zancudomyces culisetae)
Source: Sci Rep. 2020 Jul 30;10:12886. doi: 10.1038/s41598-020-69828-9 (PMC7393158; doi:10.1038/s41598-020-69828-9)
Supplement: Supplementary file 2 — Supplementary Figures [file 41598_2020_69828_MOESM2_ESM.docx]

**Title:** Shifts in the Microbiota Associated with Male Mosquitoes (*Aedes aegypti*) Exposed to an Obligate Gut Fungal Symbiont (*Zancudomyces culisetae*)

**Author:** Jonas Frankel-Bricker^1*^

**Author affiliation:**

^1^Department of Biological Sciences, Boise State University, Boise, Idaho 83725

***Corresponding Author email:** jonasfrankelbricker@gmail.com

**Author ORCID:** 0000-0001-7630-9002

**Running title:** Male mosquito microbiota

**Keywords:** microbiome, mosquitoes, host-microbe interactions, gut fungi, Aedes aegypti, *Zancudomyces* *culisetae*


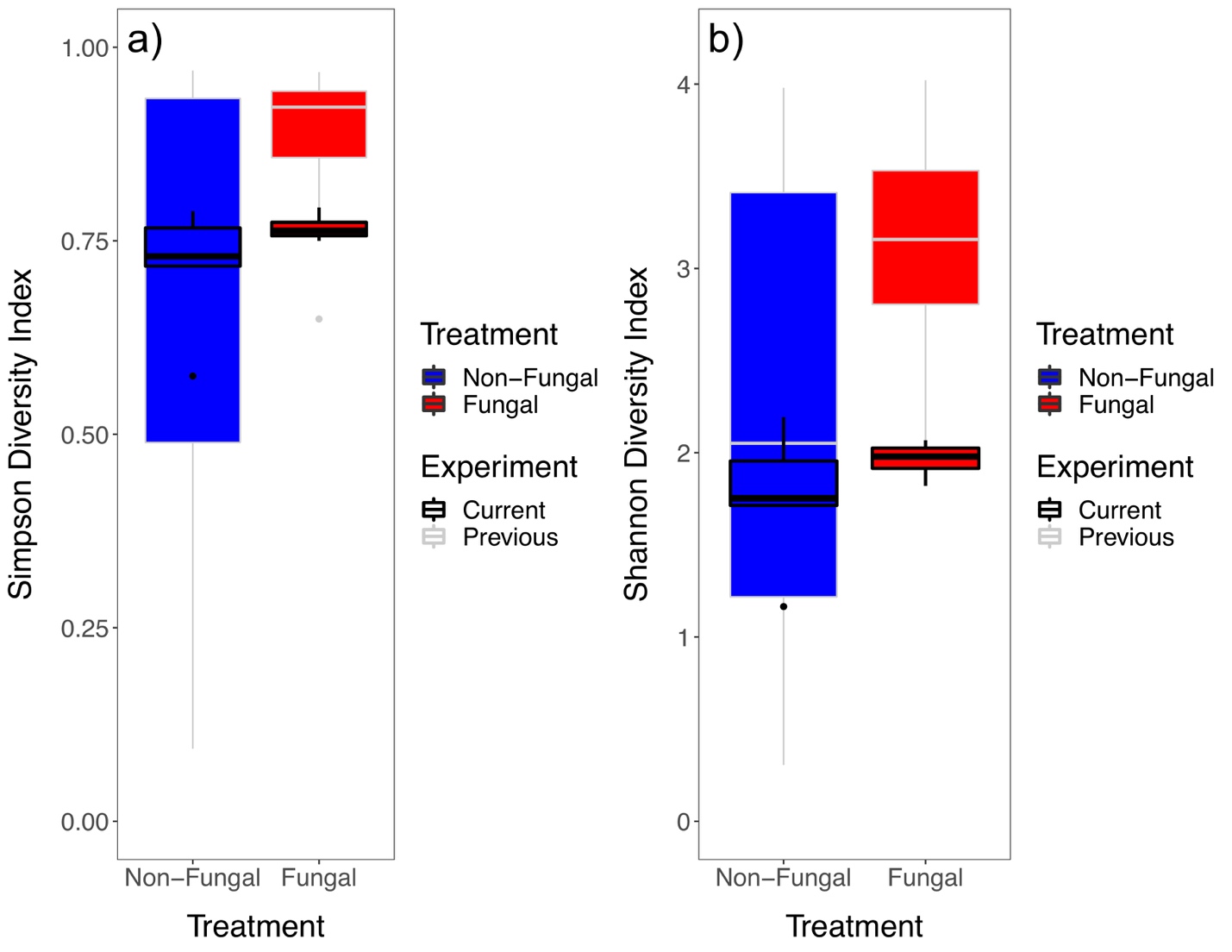


**Fig. S1** Comparative box plots of alpha diversity measures of microbiomes harbored in newly emerged adult females that were frozen prior to DNA extraction (Current, *N* = 9) and samples previously reported in Frankel-Bricker et al., 2020 (Previous, *N* = 46) for (**a**) Simpson and (**b**) Shannon diversity indices.


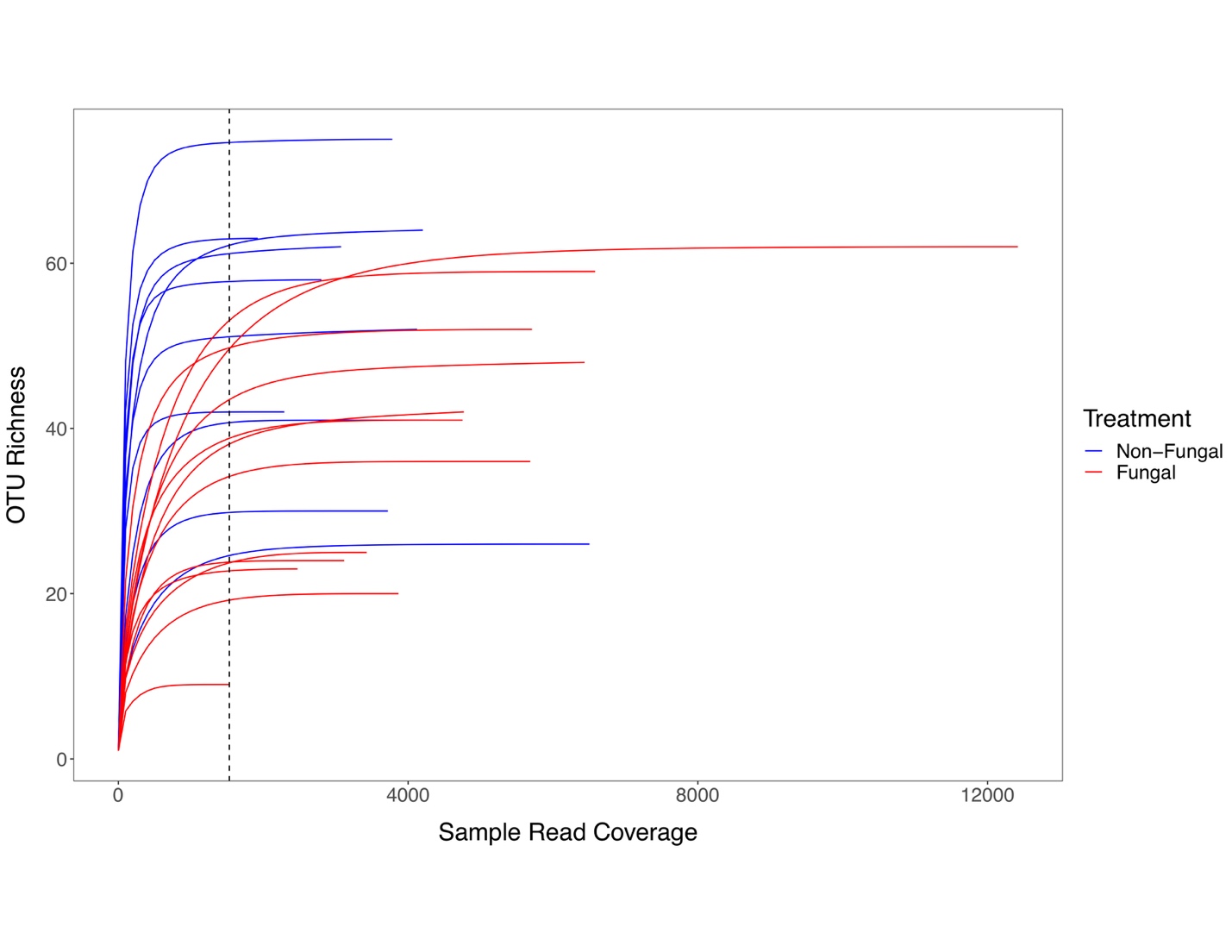


**Fig. S2** Rarefaction curves for sequencing reads. The read cutoff value is indicated with a vertical dashed line (1532 reads).
